# Examining Medical Staff Well-Being through the Application and Extension of the Job Demands–Resources Model: A Cross-Sectional Study

**DOI:** 10.3390/bs13120979

**Published:** 2023-11-28

**Authors:** Tiantian Jing, Xiaoyan Li, Chenhao Yu, Mayangzong Bai, Zhiruo Zhang, Sisi Li

**Affiliations:** School of Public Health, Shanghai Jiao Tong University School of Medicine, Shanghai 200025, China

**Keywords:** work motivation, regulatory focus, promotion focus, prevention focus, job satisfaction, emotional exhaustion

## Abstract

For medical staff, job satisfaction is essential for advancement on an individual and organizational level. This study looked into the relationships between challenging job demands, job resources, personal resources, and well-being. Additionally, it examined the potential mediating effects of emotional exhaustion and work motivation within the framework of the job demands–resources (JD–R) model. Results from a cross-sectional study of 267 medical employees at a second-grade comprehensive hospital in Jiangsu, China’s mainland, indicated that challenging job demands and job satisfaction were positively correlated and mediated via (decreasing) emotional exhaustion. The relationship between job resources and job satisfaction was found to be mediated via (decreasing) emotional exhaustion and (increasing) work motivation. The investigation also demonstrated that the two regulatory focuses serve different purposes. It was discovered that promotion focus had a favorable effect on work motivation but a negative effect on emotional exhaustion. Conversely, preventive focus only positively predicted emotional exhaustion. Thus, the JD–R model offers a valuable structure for clarifying the job satisfaction of health personnel. The implications for enhancing individual and job outcomes are discussed.

## 1. Introduction

Job satisfaction plays a critical role in healthcare organizations, the high expenses associated with the workforce, and the widespread scarcity of staff [1]. However, it is widely acknowledged that work-associated dissatisfaction is prevalent among health personnel worldwide [2,3], and this situation has also been observed in China [4,5]. The well-being of healthcare professionals has garnered significant attention in light of their demanding roles and the increasing demands on medical services, particularly in developing countries [6] and during the COVID-19 pandemic [7]. Job satisfaction is a worldwide worry because of its possible influence on the quality and security of patient treatment. Furthermore, it has been discovered that a lack of job satisfaction can lead to decreased dedication to the organization, diminished job effectiveness, and ultimately, individuals leaving their current jobs and the field [8,9,10,11]. Considering the challenges faced in medical settings, it is crucial to comprehend the factors that impact employees’ job satisfaction.

The job demands–resources (JD–R) model [12] provides a framework for studying medical staff [13]. Expanding on this model, job demands encompass both challenges and hindrances [14]. Challenging job demands exhibit duality, as they can both deplete energy and stimulate individuals because of their perceived instrumental role in achieving valued outcomes, such as work goal attainment [14]. Incorporating individual traits into the JD–R model was one of the aspects of our investigation, as the comprehension of human behavior relies on the dynamic interplay between personal traits and working environment elements [15]. Numerous scholarly investigations have assessed the influence of individual characteristics on the overall well-being of employees by applying the JD–R model. A recent review identified four distinct approaches for integrating personal resources into the JD–R model. Among these approaches, it was found that regulatory focus acted as a precursor to both strain and motivation [16,17]. 

Our study aimed to achieve three primary objectives based on the JD–R model. First, incorporating patient-centered care (PCC) as a challenging job demand was prioritized, acknowledging its significance within the medical context. Second, we examined the impact of challenging job demands (PCC) and job resources (psychological safety) on job satisfaction among health personnel in China, with emotional exhaustion and work motivation serving as mediators. Moreover, the theoretical model of the JD–R framework is enhanced by incorporating regulatory focus as personal resources. Specifically, our study delved into the association between patient-centered care, psychological safety, promotion and prevention focus, and job satisfaction.

## 2. Theoretical Background

### 2.1. Job Demands–Resources Model

The job demands–resources (JD–R) model serves as a comprehensive framework applicable to various job-related contexts [18,19]. Examining the associations between diverse job demands and resources within organizations across different nations is significant because of potential variances.

Work environment characteristics can be classified into two categories based on the premise that each profession entails distinct risk factors associated with stress from occupation, namely job demands and job resources [12]. The former include components of a job that require continuous physical and/or psychological effort, resulting in physiological and/or psychological consequences. The latter refers to the physical, social, or organizational characteristics of a job that contribute to the achievement of work-related objectives, lessen the negative impact of workplace demands on individuals’ outcomes, and encourage personal growth and development. Despite the conceptual differentiation between job demands and resources, job demands have traditionally been perceived as unfavorable physical, social, or organizational facets, while job resources have been viewed in a positive light [15]. 

The JD–R model also emphasizes the involvement of two distinct psychological mechanisms that contribute to job stress and motivation [20]. The first is the health impairment process, wherein high job demands can potentially result in burnout by depleting employees’ cognitive and physical energy, consequently giving rise to health-related issues [21,22]. The second is the motivational process, in which job resources act as (intrinsic and extrinsic) motivators to augment work engagement and facilitate exceptional performance, thereby fostering the commitment and achievement of work-related objectives [18]. The research findings indicate that the JD–R model does not establish a direct connection between job demands and motivational outcomes. However, job resources are directly associated with outcomes pertaining to health impairment and motivational processes [15,23].

### 2.2. Challenge Job Demands and Well-Being

Despite extensive research on the JD-R model, recent reviews have carried out various concerns [24,25]. The first weakness pertains to the conceptualization of job demands. Job resources are widely recognized to have a direct relationship with positive indicators of the well-being of individuals in relation to their work. Conversely, the associations between job demands and well-being are contingent on the specific nature of the demand [14,26]. Cavanaugh et al. attempted to distinguish two types of demands: hindrances, which impede the achievement of goals, and challenges, which aid in the achievement of goals [27]. Employees perceive hindrances to job demands as a significant obstacle that drains their energy, leading to a sense of limited control and the experience of negative emotions [14]. In contrast, challenging job demands possess a dual nature in their characteristics. They can be both energy-depleting and stimulating at the same time, as they are perceived as instrumental in attaining valued outcomes, such as work goal achievement [26]. Instances of challenge demands consist of heightened levels of job responsibility, time urgency, and heavy workloads. Employees may perceive these demands as opportunities for learning, accomplishing tasks, and receiving rewards [28].

Guided by the JD–R model, we posit that patient-centered care (PCC) poses a challenging job demand in the context of Chinese medical practice. PCC encompasses the acknowledgment and adaptation of individual patients’ care needs, preferences, and principles in every clinical decision-making procedure [29]. On the one hand, PCC mandates that health personnel possess robust interpersonal communication and emotional perception skills. In the healthcare field, PCC prioritizes medical interviews and requires the application of procedural, cognitive, and affective skills [30]. Interpersonal demands and perceptual skills play a crucial role in clinical reasoning and problem-solving throughout the PCC process. However, possessing both skill sets also encompasses interpersonal competencies that can significantly augment communication. It is important to acknowledge that these requirements are under the purview of health personnel who have the capacity to nurture their education, personal development, and accomplishments within the professional setting, thereby cultivating a sense of personal satisfaction and professional gratification [31]. Evidence from various healthcare settings indicates that the quality of patient–provider relationships, specifically those focused on actively engaging patients in their healthcare pathway, is associated with favorable outcomes for both patients and providers [32]. Therefore, we propose the following hypothesis:

**Hypothesis** **1.** *Challenge job demands (i.e., PCC) of medical staff are negatively related to emotional exhaustion.*

### 2.3. Job Resources and Well-Being

Researchers have examined job resources encompassing various aspects of a job, leading to their classification at multiple levels [33]. Job resources can be found at various levels, including the organization, interpersonal and social relations, work-related factors, and the task itself [20]. This suggests that in organizations (e.g., medical environments) with high complexity and dynamics, the organization and management’s implementation and execution of a safety atmosphere and procedures can be perceived as a valuable resource for employees in coping with the effects of dynamics.

Psychological safety, a prominent psychological job resource, is of considerable significance in medical contexts. In a work environment characterized by intricacy and dynamism, health personnel must collaborate independently to ensure the provision of secure patient care [34]. Psychological safety in the workplace refers to the collective belief among individuals about the safety of taking interpersonal risks [35,36]. Psychological safety, a valuable psychological asset, has the potential to foster an environment in which employees feel comfortable expressing their opinions, trusting and respecting their colleagues, and mitigating their fear of failure. Consequently, this can engender a heightened emotional affinity toward the organization and a notable enhancement in employees’ overall well-being [37,38]. The presence of a psychologically safe working environment motivates employees to devote more attention and resources to improving their performance, thereby fostering heightened levels of work motivation [39]. Moreover, psychological safety plays a pivotal role in assisting individuals to overcome anxiety and defensiveness, augment their self-awareness, regulate their emotional responses, and avert emotional exhaustion [40,41]. Conversely, those who are psychologically unsafe may be reluctant to seek assistance when necessary or may choose to remain silent upon identifying a problem, thereby diminishing the likelihood of positive emotional experiences and heightening the risk of emotional depletion [42]. Another way in which psychological safety can also affect well-being is by facilitating knowledge-sharing processes. Individuals who possess a heightened sense of psychological safety are more likely to engage in constructive debates and discussions, thereby establishing clear expectations from each other [43]. Based on this, we hypothesized the following:

**Hypothesis** **2.** *The job resources (i.e., psychological safety) among medical staff exhibit a positive correlation with work motivation and a negative correlation with emotional exhaustion.*

### 2.4. Personal Resources and Well-Being 

Personal resources were employed as antecedents to investigate the direct influence of personal resources on job satisfaction, while also considering the support provided by the existing literature [15]. As previously mentioned, our intention was to incorporate regulatory focus as a personal resource within the JD–R model and subsequently examine its influence on the well-being of health personnel.

According to the regulatory focus theory (RFT), human behavior is influenced by two distinct approaches: promotion and prevention focus [44]. Promotion-focused individuals are motivated to achieve their “ideal selves” through goals, ideals, and accomplishments and are more attuned to positive outcomes. Conversely, prevention-focused individuals prioritize their “ought selves” characterized by duties, obligations, and responsibilities, and are more responsive to negative outcomes [45,46]. Furthermore, empirical evidence has demonstrated that promotion and prevention foci are entirely different strategies, indicating that an individual may concurrently exhibit elevated levels of both foci, exclusively possess one focus, or lack either focus [47]. Lanaj et al. (2012) posited that individuals emphasizing promotions are inclined to be more attuned to the favorable aspects of their work, which, in conjunction with their proclivity toward positive overall sentiments and heightened emotional well-being, predisposes them to experience higher job satisfaction. Individuals who place a higher emphasis on prevention tend to focus on the negative aspects of their work, resulting in the reception of unfavorable messages. Empirical research supports these claims, demonstrating that promotional focus is positively associated with job satisfaction, engagement, affective commitment, task performance, and organizational citizenship behavior. In contrast, prevention focus exhibited a negative correlation with job satisfaction, displayed no significant association with task performance, and demonstrated a positive relationship with emotional exhaustion [45,48,49]. Comparable findings were obtained through research conducted in a medical setting. For example, a longitudinal study indicated that prevention focus was associated with an accelerated build-up of health impairment outcomes, while promotion focus was linked to an accelerated build-up of motivational outcomes [50]. In a separate study, Koopmann et al. examined the distinct roles of regulatory foci in predicting helping and voice behaviors through mediating factors of emotional exhaustion [51]. In the Chinese medical context, Shi et al. revealed that a promotion focus was found to decrease burnout when participants perceived transformational leadership. Conversely, a prevention focus displayed the opposite pattern [52]. Thus, the following hypothesis is proposed:

**Hypothesis** **3.** *The promotion focus among medical staff exhibits a positive correlation with work motivation and a negative correlation with emotional exhaustion.*

**Hypothesis** **4.** *The prevention focus among medical staff exhibits a negative correlation with work motivation and a positive correlation with emotional exhaustion.*

### 2.5. Emotional Exhaustion, Work Motivation, and Job Satisfaction

Emotional exhaustion refers to a state of mental exhaustion in which an employee’s emotional and energetic resources are depleted [53]. It functions as a crucial component of the burnout construct, which is distinguished by cynicism and reduced effectiveness [54]. Butkus and Green have defined work motivation as “persuade, push, and move to act in accordance to needs satisfaction” [55]. 

An examination of the JD-R model demonstrated that emotional exhaustion and work motivation are important mediators in the JD–R model, connecting job characteristics and well-being [18,56]. Subsequent studies [57,58,59] have yielded additional empirical support for the assertion that job demands are indicative of employee performance via emotional exhaustion, and job resources have been identified as positive influencers of performance through work motivation. Furthermore, job resources and emotional exhaustion are cross-linked. Indeed, a study conducted on university faculty supported the JD–R model by demonstrating that high job demands, such as work–family conflict, were a significant predictor of low job satisfaction through the pathway of emotional exhaustion. On the other hand, job resources, such as leader support, were found to be predictors of high job satisfaction through the pathway of work engagement [60].

Finally, considering that personal resources have a similar effect as job resources in the JD–R model [61], we propose that the effect of personal resources (i.e., promotion and prevention focus) may be mediated by emotional exhaustion and work motivation. According to a recent review, individuals with positive personal resources have been found to have a strong connection with work engagement and are less susceptible to burnout. These individuals are characterized by their openness, warmth, supportiveness, and perseverance. However, individuals with negative personal resources are more prone to experiencing burnout. This can be attributed to their inclination to focus on the negative aspects of a situation and their tendency to remember negative information about the situation afterward. This negative cognitive bias can contribute to a heightened sense of exhaustion, cynicism, and inefficacy, ultimately leading to burnout [24]. In accordance with the JD–R model, this study aimed to examine the potential indirect association between challenge job demands (PCC), job resources (psychological safety), and personal resources (promotion and prevention focus) with job satisfaction, mediated by emotional exhaustion and work motivation.

**Hypothesis** **5.** *Emotional exhaustion among medical staff serves as a mediator between challenging job demands (PCC) and job satisfaction.*

**Hypothesis** **6A.** *Emotional exhaustion among medical staff acts as a mediator in the relationship between job resources (psychological safety) and job satisfaction.*

**Hypothesis** **6B.** *Work motivation among medical staff acts as a mediator in the relationship between job resources (psychological safety) and job satisfaction.*

**Hypothesis** **7.** *The relationship between personal resources (promotion and prevention focus) and job satisfaction is mediated by both emotional exhaustion and work motivation among medical staff.*

### 2.6. The Present Study

**Figure 1:**
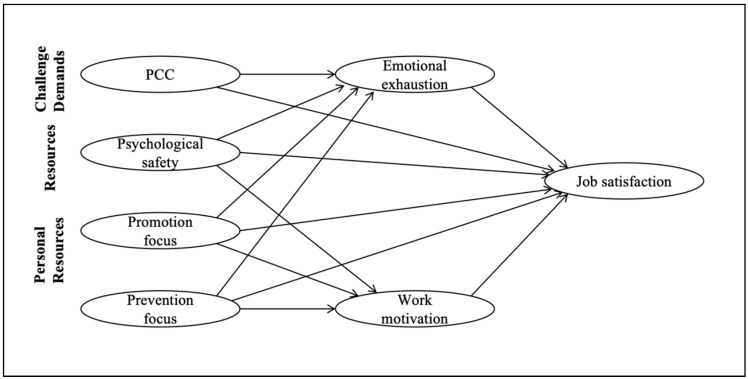
Theoretical model. Note: PCC, patient-centered care.

Accordingly, the present study investigated whether the relationship between challenging job demands (PCC), job resources (psychological safety), personal resources (promotion and prevention focus), and job satisfaction of Chinese medical staff is mediated by emotional exhaustion and work motivation. The hypothesized model is summarized in Figure 1.

## 3. Materials and Methods

### 3.1. Participants

Convenience sampling was conducted at a second-grade comprehensive hospital in Jiangsu, China. This hospital has many medical departments and is representative of hospitals of this type in China. Ethical approval for this study was obtained from (university withheld for blinded review) (SJUPN-202104). Consent to participate was collected at the beginning of the survey. An anonymous online survey (see Section 3.2) was administered to all medical employees at the hospital in September 2021 (*n* = 334). A total of 267 valid responses were obtained (response rate = 79.94%) from respondents who were mainly female (68.2%) with bachelor’s degrees (76.8%), and their average age was 32.30 years (SD = 6.34). Of the participants, 74.2% were physicians or nurses (Table 1). 

### 3.2. Measures

A battery of existing questionnaires with appropriate psychometric properties were used. To ensure linguistic accuracy, we followed Brislin’s “back translation” procedures when translating the scales from English to Chinese, as they were originally developed in English [62]. Two senior postgraduate Public Health students translated the original English scales into Chinese, and two senior postgraduate students backtranslated the Chinese versions into English. Consistency and accuracy were checked by a social psychologist, and several experts from relevant disciplines checked the translated questionnaires to ensure that they were suitable for the Chinese medical environment. We calculated the average scores for each scale (or dimension, if applicable), with higher scores indicating greater levels of the construct under investigation. 

Emotional exhaustion. The 9-item Emotional Exhaustion subscale from the Maslach Burnout Inventory–Human Services Survey (MBI-HSS) [63,64] was used. This scale has been extensively employed in occupational psychology owing to its favorable psychometric properties across diverse sociocultural contexts [65]. Responses were recorded on a 7-point Likert scale (1 = never to 7 = every day). Examples of items include “I feel emotionally drained from my work”. 

Work Motivation. The 12-item Motivation-at-Work Scale (MAWS [66]) was also used. Responses were recorded on a 7-point Likert scale (1 = completely disagree to 7 = completely agree). Examples of items include “I do this job because I have fun doing my job” (intrinsic motivation); “I chose this job because it allows me to reach my life goals” (identified regulation); “Because my work is my life and I don’t want to fail” (introjected regulation); and “I do this job because this job affords me a certain standard of living” (external regulation). 

Regulatory Focus. The 18-item Regulatory Focus Questionnaire (RFQ [67]) was used. Responses were rated on a 5-point Likert scale (1 = completely disagree to 5 = completely agree). Examples of items include “I typically focus on the success I hope to achieve in the future” (promotion focus) and “I frequently think about how I can prevent failures in my life” (prevention focus). 

Patient-centered care. The 16-item Patient–Professional Interaction Questionnaire (PPIQ) [68] was used by changing statements to reflect the medical staff’s perspectives. Responses were rated on a 5-point Likert scale (1 = not at all to 5 = very much). Examples of items include “I provided my patients with clear information” (effective communication); “I was interested in what my patients know about their disease/prognosis” (interest in patient’s agenda); “I understood my patients’ emotions” (empathy); and “I gave my patients time to ask and to talk about the disease” (patient involvement in care).

Psychological safety. Three items adapted from Detert and Burris were used for evaluation [35,69]. Responses were rated on a 7-point Likert scale (1 = very inaccurate to 7 = very accurate). Examples of items include “In this hospital, it is safe for me to make suggestions”. 

Job Satisfaction. The short form of the Minnesota Satisfaction Questionnaire [70] was used. Responses were rated on a 5-point Likert scale (1 = very dissatisfied to 5 = very satisfied). Examples of items include “I have the chance to do something that makes use of my ability”. 

Demographic (e.g., age, sex, and educational level) and occupation-related data (e.g., shifts, employment type, and level) were also collected. 

### 3.3. Analytical Plan

All analyses were conducted using SPSS AMOS 24.0, IBM SPSS 26.0 (Statistical Package for the Social Sciences, IBM Corp., Armonk, NY, USA) [71,72], and R 4.2.1 [73].

The reliability and validity of the translated scale were examined before hypothesis testing. Cronbach’s alpha was used to evaluate the internal reliability of the scales; values > 0.60 were deemed satisfactory [74]. A confirmatory factor analysis was performed to examine the discriminant validity of the scales. The square root of the average variance extracted for each latent factor must exceed the correlation between that factor and other latent factors [75]. As recommended, the quality of fit of the measurement model was first evaluated using confirmatory factor analysis (CFA), and then the structural model was validated for hypothesis testing [76]. To assess the goodness of fit, we evaluated the *χ*^2^/*df* value and fit indices, which met standard criteria such as the Comparative Fit Index (CFI) and Tucker–Lewis index (TLI) exceeding 0.90, and the Root-Mean-Square Error of Approximation (RMSEA) below 0.08 [77,78,79,80]. The variance inflation factor (VIF) was used to assess multicollinearity. Considering the cross-sectional design, a predetermined stringent threshold was established, whereby a VIF value below 2.5 was deemed as acceptable [81].

## 4. Results

### 4.1. Intercorrelations, Statistical Means, and Standard Deviations

Descriptive statistics, average variance extracted, and correlations of the main variables are summarized in Table 2. All scales showed satisfactory content and discriminant validity, as the square root of the AVEs was larger than all correlations in the rows and columns. As expected, PCC, psychological safety, work motivation, and promotional focus were positively related to job satisfaction, whereas emotional exhaustion was negatively related to job satisfaction.

### 4.2. Assessment of the Measurement Model

A confirmatory factor analysis was used to test the measurement model, which consisted of seven latent variables. The findings indicated that the model adequately fit the collected data: *χ*^2^/*df* = 1.711, *p* < 0.001, CFI = 0.906, TLI = 0.902, SRMR = 0.073, and RMSEA = 0.052. We compared the measurement model to another alternative model, which created one factor for regulatory focus (*χ*^2^/*df* = 2.007, *p* < 0.001, CFI = 0.866, TLI = 0.861, SRMR = 0.075, RMSEA = 0.062), and the *χ*^2^ difference between the two models was significant (*χ*^2^*_difference_* (23) = 875.7, *p* < 0.001).

### 4.3. Assessment of Common Method Variance

Several precautions were taken to minimize the common method variance (CMV). First, in the introductory section of the questionnaire, we explicitly assured participants that the absence of a definitive correct or incorrect response was ensured. Secondly, we randomly ordered the items within each scale to reduce the priming effect of item embedding in the question context. Statistical tests were conducted to determine the extent of CMV infection. The results of Harmon’s one-factor test showed that the single factor extracted accounted for 36.693% of the variance, which is below the criterion of 50% [82,83]. Meanwhile, testing a one-factor model showed poor fitting indices: *χ*^2^/*df* = 5.206, *p* < 0.001, CFI = 0.439, TLI = 0.424, SRMR = 0.165, and RMSEA = 0.126. Therefore, CMV did not pose a threat to the data. 

### 4.4. Assessment of the Structural Model

**Figure 2:**
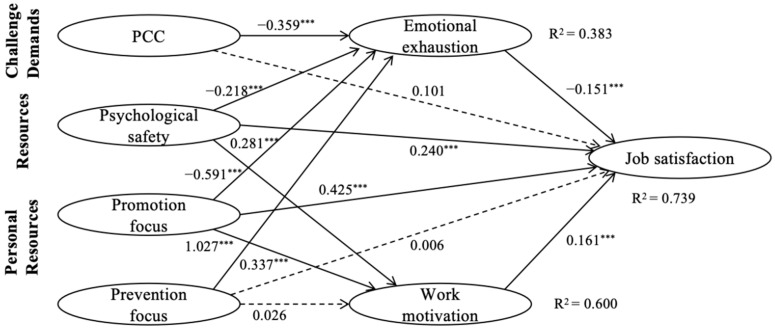
Results of the structural model. Note: Standardized coefficients are reported. The solid lines indicate significant coefficient paths and the dotted lines indicate nonsignificant coefficient paths. *** *p* < 0.001.

The structural model also showed a good model fit (*χ*^2^/*df* = 2.386, *p* = 0.003, CFI = 0.976, TLI = 0.940, SRMR = 0.025, RMSEA = 0.072), and the variance explained for each of the endogenous variables is 38.3% for emotional exhaustion, 60.0% for work motivation, and 73.9% for job satisfaction. Given that the highest variance inflation factor (VIF) score was 2.46, multicollinearity was not considered problematic. The standardized coefficient paths are shown in Figure 2. The figure showed that PCC (*β* = −0.359, *p* < 0.001), psychological safety (*β* = −0.218, *p* < 0.001), and promotion focus (*β* = −0.591, *p* < 0.001) negatively predicted emotional exhaustion, while prevention focus (*β* = 0.337, *p* < 0.001) positively predicted emotional exhaustion. Psychological safety (*β* = 0.281, *p* < 0.001) and promotion focus (*β* = 1.027, *p* < 0.001) positively predicted work motivation, while prevention focus (*β* = 0.026, *p* = 0.066) was nonsignificant for work motivation. Finally, work motivation (*β* = 0.161, *p* < 0.001), psychological safety (*β* = 0.240, *p* < 0.001), and promotion focus (*β* = 0.425, *p* < 0.001) were found to have a direct and positive effect on job satisfaction, while emotional exhaustion (*β* = −0.151, *p* < 0.001) was found to have a direct and negative effect on job satisfaction. 

### 4.5. Assessment of the Mediation Hypotheses

We estimated the degree of significance of the indirect effects of emotional exhaustion, work motivation, and job satisfaction using a bootstrap approach. Based on 5000 bootstrap data samples, we observed a positive indirect effect (0.054; 95% CI = 0.021–0.101) of PCC on job satisfaction through emotional exhaustion. Similarly, the indirect effects of psychological safety on job satisfaction via emotional exhaustion (0.033; 95% CI = 0.014–0.059) and work motivation (0.045; 95% CI = 0.019–0.081) were also significant. Moreover, the indirect effect of promotion focus on job satisfaction via emotional exhaustion (0.089; 95% CI = 0.043~0.148) and work motivation (0.165; 95% CI = 0.085~0.245) was significant, while only prevention focus on job satisfaction via emotional exhaustion (−0.051; 95% CI = −0.086~−0.025) was significant. 

### 4.6. Multi-Group Analyses

Multi-group analyses were performed to examine the model invariance. The results revealed that prevention focus was positively related to physicians’ work motivation. It is worth mentioning the negative effect of PCC on emotional exhaustion in terms of the magnitudes for nurses than for physicians. However, emotional exhaustion had only a mediating effect on physicians (Supplementary Figure S1). In another test, the model behaves similarly for both employment types (Supplementary Table S1 and Figure S2).

## 5. Discussion

### 5.1. Interpretation of Results

Our research yielded empirical evidence supporting the applicability of the JD–R model to medical contexts in China. Specifically, we focused on exploring the dual processes outlined in the JD–R model and assessed the influence of challenging job demands and personal resources on medical staff. Notably, we discovered a positive relationship between challenging job demands (i.e., PCC) and job resources (i.e., psychological safety) on the well-being of medical staff, which holds significance. Additionally, personal resources (i.e., regulatory focus) were found to have a direct and important impact on the JD–R model (supporting Hypotheses 1–3 and partly supporting Hypothesis 4). The findings of this study support the hypothesized mediators, namely emotional exhaustion and work motivation, indicating that both job and personal characteristics can impact employees’ job satisfaction by not only reducing negative emotional assessments but also enhancing positive ones (supporting Hypotheses 5 and 6, partly supporting Hypothesis 7) [24,84,85]. Additionally, our observations indicate that job resources exert a more substantial impact on work motivation than emotional exhaustion [86]. Emotional exhaustion and work motivation had comparable influences on job satisfaction.

It was found that PCC had an indirect impact on job satisfaction, as it primarily influenced emotional exhaustion. The direct effect of PCC on job satisfaction was not statistically significant, indicating full mediation. Challenging job demands tend to generate positive emotions, which are responses to events that indicate achievement and progress toward a favorable outcome. In addition, the successful handling of work stress generates positive emotions, such as self-worth and achievability [87]. Challenging job demands and emotional evaluation (emotional exhaustion) were more closely related than cognitive evaluation (job satisfaction). A study cautioned that the assessment of challenging job demands may vary over time [28]. Similarly, Tadić et al. (2014) argue that the classification of job demands is contingent on the nature of the demand [88]. For instance, a physician may begin to think of PCC as a barrier rather than a challenge.

Our study explored the predictive effects of two regulatory foci (promotion focus and prevention focus) on the well-being of medical staff that have received limited attention in the JD–R model antecedents. Interestingly, the findings revealed a noteworthy positive association between promotion focus and job satisfaction mediated by emotional exhaustion and work motivation. Conversely, a prevention focus alone negatively predicted emotional exhaustion. This inconsistency can be interpreted within the framework of the conservation of resources theory, as promotion-focused strategies have the potential to serve as resources that can aid in coping with threats [52].

The mediation effects were similar for permanent and contact-based employees. Path analyses revealed that PCC decreased emotional exhaustion to a greater extent in nurses than in physicians. This finding can be attributed to the fact that nurses typically assume the responsibility for delivering daily and continuous patient care, which results in more frequent and intimate interactions with patients. Consequently, nurses were more likely to perceive the direct impact of their actions and experience immediate feedback, leading to a greater sense of satisfaction. Conversely, physicians must consider the effectiveness and prognosis of patients when providing PCC, thereby diluting its impact on emotional well-being.

### 5.2. Implications, Limitations, and Conclusions

#### 5.2.1. Theoretical Implications

Previous studies have demonstrated a significant and extensive array of psychological consequences stemming from the COVID-19 pandemic in health personnel, manifesting as elevated levels of psychological symptoms, such as anxiety, depression, somatic symptoms, and burnout [89,90]. Unlike the application of the JD–R model in the COVID-19 period, where job demands and resources mainly focus on pandemic-related tasks, such as resource allocation, coordinating patient and family needs, caring for critically ill patients, and balancing the well-being of healthcare workers, our study examines the application of the JD–R model in a regular medical work environment [91,92]. The theoretical contributions of this study are as follows.

First, our theoretical model successfully supported our hypotheses; that is, in the Chinese medical setting, PCC acts as a challenging job demand, as it has duality as a job demand and the potential for future growth and attainment. Because individuals tend to assess challenging job demands as opportunities to grow, learn, and achieve goals, they often strive to achieve the opportunities available under challenging stress. Our findings support Schaufeli and Taris (2014) in that challenging job demands can yield positive and valuable outcomes that surpass the perceived stress induced by stressors [15]. Consequently, this study expands on prior research on the JD–R model and contributes to its ongoing advancement by examining the notion of challenging job demands in a Chinese medical context.

Second, it provides valuable insights into the significance of job resources in relation to job satisfaction among medical staff. Psychological safety is recognized as a crucial determinant as it exhibits a negative correlation with emotional exhaustion and a positive correlation with work motivation. Establishing a working environment that encompasses psychological safety is imperative for individuals to experience a sense of security, enabling them to exchange ideas effectively and enhancing productivity within the workplace, thereby fostering organizational growth. Furthermore, the presence of psychological safety contributes to heightened job performance, as employees who feel secure exhibit increased levels of engagement in their professional responsibilities [93]. Our research discovered that psychological safety could serve as a job resource within the JD–R model, leading to enhanced job satisfaction through a reduction in emotional exhaustion and amplification of work motivation. Consequently, our findings contribute to the existing body of literature and may inspire researchers to incorporate psychological safety as a job resource when considering the predictors influencing the well-being of medical staff at the individual level. 

Third, although the inclusion of personal resources in the JD–R model is widely acknowledged, there is a divergence of opinions regarding the specific role these resources play within the model, whether as antecedents, mediators, moderators, “third variables”, or a combination thereof [15]. Our study demonstrates that regulatory focus, as a personal resource, functions as an antecedent in the JD–R model. Consequently, this study contributes to establishing a foundation for understanding the significance of personal resources in the JD–R model.

#### 5.2.2. Practical Implications

The current investigation indicated that the overall job satisfaction of the Chinese medical staff slightly exceeded the moderate attitude. However, decreased job satisfaction negatively impacts staff retention, healthcare quality, and patient outcomes [94]. Our study offers valuable practical insights for enhancing job satisfaction among Chinese medical staff.

The present findings offer practical insights for hospital human resource management in hospitals. First, given the challenging job demands (PCC) and their positive relationship with medical staff well-being, it is advisable to motivate health personnel to perceive PCC as an opportunity rather than an obstacle. Instrumentally, hospital administrators should prioritize the establishment and sustenance of a positive and reliable healthcare environment, as the foundation of trust between physicians and patients relies on patients’ experiences and perceptions of the hospital and healthcare as a whole. Medical staff, especially physicians, can enhance PCC through interventions, such as training programs, that improve emotional management and communication skills. Furthermore, they should prioritize intrinsic satisfaction, such as professional pride, to further improve healthcare delivery. 

Second, owing to the changeable assessment of challenging job demands, our findings additionally indicate the significance of psychological safety for medical staff. To enhance staff psychological safety, hospitals may consider implementing strategies such as adopting democratic and supportive management styles [34], promoting seeking work-related support among staff, and fostering collaborative work environments.

Finally, regulatory focus has a direct impact on well-being by translating tendencies into behaviors. Consequently, well-being can be promoted by fostering the adoption of promotion-focused strategies among medical staff and their administrators during goal pursuit [52]. As mentioned above, regulatory focus serves as a motivational variable and a proximal factor in work outcomes [45]. Encouraging individuals to utilize promotion-focused rather than prevention-focused strategies is more likely to result in heightened well-being. 

### 5.3. Study Limitations and Future Direction

The following limitations of this study are acknowledged. First, the cross-sectional design employed hindered the ability to establish causal relationships. Second, despite achieving a questionnaire response rate of approximately 80%, the potential for non-response bias exists because of the survey’s brevity and uninterrupted completion time of 30 min, despite prior communication with hospital administrators regarding the investigation. Third, it is noteworthy that PCC and the job satisfaction measurement tool exhibited a relatively high Cronbach’s alpha value, which suggests that future studies might adopt different measurement tools to replicate the current findings and generalize their implications. Fourth, it is worth noting that the previous literature has extensively investigated various aspects of personal resources within the JD–R model. However, our study focused solely on personal resources as antecedents. Future studies should investigate additional potential functions, such as the mediation or moderation of personal resources, within the JD–R model, supported by empirical data. Finally, data were collected from one second-grade comprehensive hospital using convenience sampling, which may have caused selection bias and generalizability and should be considered in light of this methodological constraint. Future studies should select participants from multiple levels and types of hospitals. 

Future research would benefit from utilizing longitudinal study designs to track the effects of challenging job demands, as well as personal resources, on individuals’ well-being. Additionally, conducting intervention studies would offer a more direct pathway to understanding the causal relationships between these variables.

This study revealed that challenging job demands (i.e., PCC) and job resources (i.e., psychological safety) could increase job satisfaction by (increasing) work motivation and (decreasing) emotional exhaustion. Additionally, personal resources, namely promotion and prevention foci, were found to act as antecedents of the JD–R model. Managers ought to demonstrate a heightened awareness of the psychological conditions prevalent among health personnel, as well as the associated influencing factors. They should implement measures aimed at mitigating emotional exhaustion, enhancing work motivation, and offering external support to bolster medical staff job satisfaction. Our findings contribute significantly to healthcare management research and practices.

## Figures and Tables

**Table 1:** Characteristics of the responding medical staff.

Demographic Characteristics	*n*	%
Gender		
Male	85	31.8
Female	182	68.2
Educational level		
Junior high school or below	2	0.8
High school or technical secondary school	3	1.1
College	38	14.2
Bachelor’s degree	205	76.8
Master’s degree or above	19	7.1
Annual income		
CNY 100,000 or less	105	39.3
Over CNY 100,000	162	60.7
Length of work (years)		
1–4	69	25.8
5–9	115	43.1
≥10	83	31.1
Shift		
Two shifts or more	180	67.4
Day shift only	87	32.6
Employment type		
Contract	102	38.2
Permanent	165	61.8
Occupation		
Physician	88	33
Nurse	110	41.2
Medical technician	41	15.4
Administration	17	6.4
Logistics	11	4.1
Level		
Other	221	82.8
Junior	6	2.3
Intermediate	10	3.8
Senior	30	11.2
	M	SD
Age (years)	32.3	6.34

**Table 2:** Descriptive statistics, correlations, reliability, and validity indexes for study variables.

Variable	Mean	SD	Cronbach’s α	1	2	3	4	5	6	7	Composite Reliability
1. PCC	4.356	0.455	0.981	(0.985)							0.996
2. Psychological safety	5.220	1.159	0.696	0.182 **	(0.530)						0.760
3. Emotional exhaustion	2.873	0.958	0.904	−0.295 **	−0.491 **	(0.526)					0.906
4. Work motivation	5.254	1.111	0.932	0.225 **	0.613 **	−0.565 **	(0.806)				0.943
5. Promotion focus	3.932	0.611	0.917	0.195 **	0.565 **	−0.456 **	0.737 **	(0.579)			0.924
6. Prevention focus	3.430	0.802	0.875	0.073	0.058	0.116	0.243 **	0.367 **	(0.460)		0.868
7. Job satisfaction	4.335	0.745	0.971	0.258 **	0.624 **	−0.628 **	0.725 **	0.739 **	0.098	(0.636)	0.972
Square root of AVE	—	—	—	0.992	0.728	0.725	0.898	0.761	0.678	0.797	—

Note. ** *p* < 0.01. PCC, patient-centered care.

## Data Availability

The original contributions presented in the study are included in the article, and further inquiries can be directed to the corresponding authors.

## Supplementary Materials

The following supporting information can be downloaded at https://www.mdpi.com/article/10.3390/bs13120979/s1, Table S1: Fit indices for the invariance test; Figure S1: Comparing the mediation effects between physicians (left panel, *n* = 88) versus nurses (right panel, *n* = 110); Figure S2: Comparing the mediation effects between permanent (left panel, *n* = 165) versus temporary (right panel, *n* = 102) staff.
