# Supplementary material for: Examining Medical Staff Well-Being through the Application and Extension of the Job Demands–Resources Model: A Cross-Sectional Study
Source: Behav Sci (Basel). 2023 Nov 28;13(12):979. doi: 10.3390/bs13120979 (PMC10741122; doi:10.3390/bs13120979)
Supplement: Supplementary file 1 [file behavsci-13-00979-s001.zip › behavsci-2698646-supplementary.pdf]

Supplemental Table S1. Fit indices for the invariance test.

Model	ΔCMIN	ΔDF	*p*	ΔGFI	ΔNFI	ΔIFI	ΔCFI
**Physicians and nurses**							
Structural weights	13.173	11	0.282	-0.013	-0.019	-0.007	-0.003
Structural covariances	16.218	15	0.368	-0.018	-0.023	-0.007	-0.002
Structural residuals	34.811	18	0.010	-0.042	-0.050	-0.032	-0.026
**Permanent and contract employees**							
Structural weights	8.968	11	0.625	-0.007	-0.010	-0.001	0.003
Structural covariances	10.883	15	0.761	-0.007	-0.012	0.001	0.005
Structural residuals	21.24	18	0.267	-0.020	-0.024	-0.008	-0.003

Supplemental Figure S1. Comparing the mediation effects between physicians (left panel, n = 88) versus nurses (right panel, n = 110).

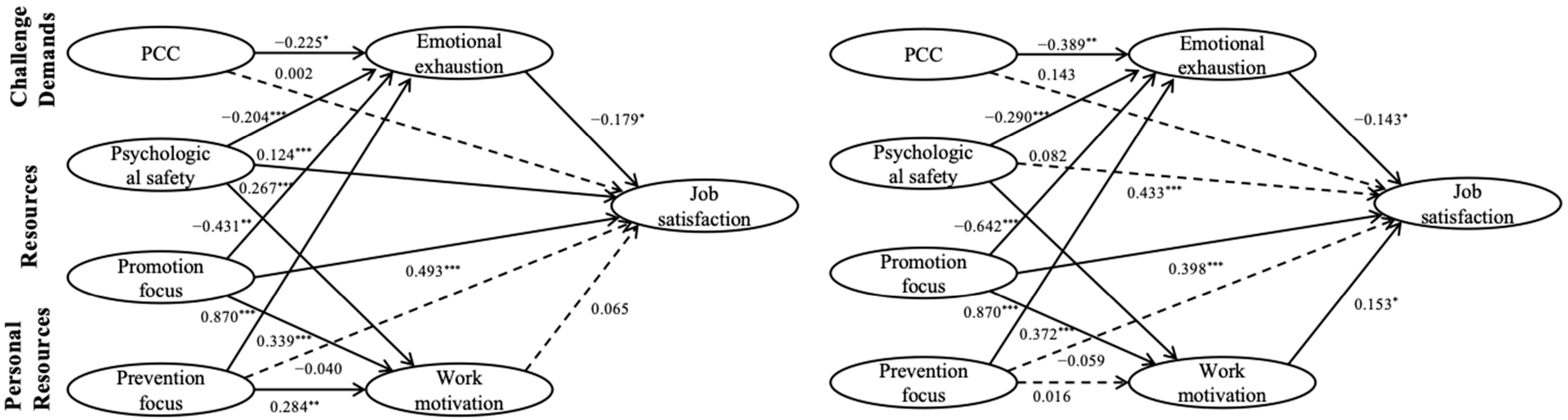

Note: Standardized coefficients are reported. The solid lines indicate significant coefficient paths and the dotted lines indicate nonsignificant coefficient paths. *** $p < 0.001$, ** $p < 0.01$, * $p < 0.05$.

Supplemental Figure S2. Comparing the mediation effects between permanent (left panel, n = 165) versus temporary (right panel, n = 102) staffs.

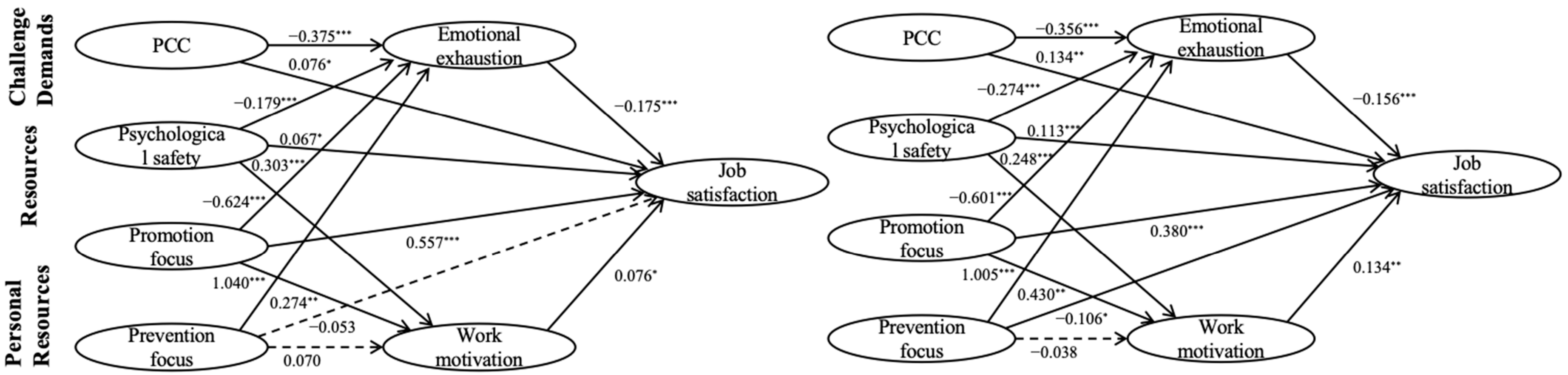

Note: Standardized coefficients are reported. The solid lines indicate significant coefficient paths and the dotted lines indicate nonsignificant coefficient paths. *** $p < 0.001$, ** $p < 0.01$, * $p < 0.05$.
